# Activity-dependent oligodendrocyte calcium dynamics and their changes in Alzheimer’s disease

**DOI:** 10.3389/fncel.2023.1154196

**Published:** 2023-10-31

**Authors:** Kenji Yoshida, Daisuke Kato, Shouta Sugio, Ikuko Takeda, Hiroaki Wake

**Affiliations:** ^1^Department of Anatomy and Molecular Cell Biology, Nagoya University Graduate School of Medicine, Nagoya, Japan; ^2^Division of Multicellular Circuit Dynamics, National Institute for Physiological Sciences, National Institute of Natural Sciences, Okazaki, Japan; ^3^Core Research for Evolutional Science and Technology, Japan Science and Technology Agency, Saitama, Japan

**Keywords:** Alzheimer’s disease, ATP, glutamate, oligodendrocyte, two photon microscopy

## Abstract

Oligodendrocytes (OCs) form myelin around axons, which is dependent on neuronal activity. This activity-dependent myelination plays a crucial role in training and learning. Previous studies have suggested that neuronal activity regulates proliferation and differentiation of oligodendrocyte precursor cells (OPCs) and myelination. In addition, deficient activity-dependent myelination results in impaired motor learning. However, the functional response of OC responsible for neuronal activity and their pathological changes is not fully elucidated. In this research, we aimed to understand the activity-dependent OC responses and their different properties by observing OCs using *in vivo* two-photon microscopy. We clarified that the Ca^2+^ activity in OCs is neuronal activity dependent and differentially regulated by neurotransmitters such as glutamate or adenosine triphosphate (ATP). Furthermore, in 5-month-old mice models of Alzheimer’s disease, a period before the appearance of behavioral abnormalities, the elevated Ca^2+^ responses in OCs are ATP dependent, suggesting that OCs receive ATP from damaged tissue. We anticipate that our research will help in determining the correct therapeutic strategy for neurodegenerative diseases beyond the synapse.

## 1. Introduction

Oligodendrocytes (OCs) form myelin around axons to regulate conduction velocity (Fields, 2008; Emery, 2010; Nave, 2010). Accumulated studies have shown the activity-dependent myelin plasticity associated with training and learning in humans and rodents (Scholz et al., 2009; Gibson et al., 2014; McKenzie et al., 2014; Xiao et al., 2016; Kato et al., 2020). Activation of neuronal activity promotes proliferation and differentiation of oligodendrocyte precursor cells (OPCs) and myelination (Wake et al., 2011; Hines et al., 2015; Mensch et al., 2015; Wake et al., 2015). Inhibition of OPC differentiation due to specific gene deletion (MyRF) in adults results in impaired motor learning process. The promotion of activity-dependent myelination increases the conduction velocity that changes the spike arrival time and contributes to temporal regulation of neuronal activity and spike timing dependent plasticity (Markram et al., 1997; Bi and Poo, 1998; Feldman, 2012). Over expression of the proteolipid protein 1 (PLP) gene results in impaired regulation of myelin basic protein (MBP) expression, which is associated with the motor learning process. Impaired activity-dependent myelination causes abnormal neuronal populational activity (increased spontaneous activity and reduced task associated activity), which ultimately results in deficient motor learning process (Kato et al., 2020).

To appropriately regulate the conduction velocity, OCs should receive information associated with neuronal impulse. Previous studies showed that OCs express receptors for neurotransmitters such as glutamate and adenosine triphosphate (ATP) and affect their metabolism (Groc et al., 2002; Agresti et al., 2005; Karadottir et al., 2005; Salter and Fern, 2005; Fields and Burnstock, 2006; Micu et al., 2006; Zonouzi et al., 2011; Fannon et al., 2015; Feng et al., 2015; Gautier et al., 2015; Spitzer et al., 2019). OPCs form a synapse-like structure with neurons, where glutamatergic signaling induces depolarization and development of Ca^2+^ transients via α-amino-3-hydroxy-5-methyl-4-isoxazolepropionic acid receptors (AMPAR) and P/Q and L type voltage-gated Ca^2+^ channels (Kukley et al., 2007; Berret et al., 2017; Barron and Kim, 2019) to promote OPC differentiation and myelination (Paez et al., 2009; Cheli et al., 2015, 2016; Barron and Kim, 2019). Optogenetically induced OC depolarization in the subiculum of the hippocampus facilitates conduction velocity in these axons, which affects the bursts of pyramidal neurons and long term potentiation (Yamazaki et al., 2019), suggesting the role of OC depolarization in glutamatergic neuronal circuitry activity. In contrast, ATP is released from presynaptic terminals via synaptic vesicles and this release is a co-release with glutamate and acetylcholine (Li and Harlow, 2014). ATP is also released from axonal segments that are situated away from the synaptic terminal (Wieraszko et al., 1989), i.e., the neuronal soma via extra synaptic vesicles. The receptors for ATP metabolism such as adenosine diphosphate, adenosine monophosphate, and adenosine expressed in OCs finally bind with P2 and P1 receptors, which show synergistic or opposing effects such as migration, proliferation, and differentiation (Stevens et al., 2002). ATP is even released in extracellular spaces of the damaged brain, suggesting that ATP contribute to the development of neurodegenerative diseases (Zelentsova et al., 2022). Myelinated axons in white matter are associated with cognitive function, and their impairment is known as Alzheimer’s disease (AD) (Amlien and Fjell, 2014). In this research, we proposed to study the differing effects of neurotransmitters on Ca^2+^ activity in OCs. Our results demonstrated that Ca^2+^ activities in OCs were differentially regulated by neuronal transmitters such as ATP or glutamate. In 5-month-old mice models of AD, larger Ca^2+^ activities in OCs were ATP dependent. These data suggest that OCs in mice models of AD mainly receive purinergic signals from the release of ATP caused by damaged tissue. This research will provide insights into the physiological and pathological responses of OCs that contribute to the pathogenesis of neurodegenerative disorders.

## 2. Materials and methods

### 2.1. Mice

All experimental protocols used in animals were approved by the Animal Care and Use Committees of Nagoya University Graduate School of Medicine and Kobe University Graduate School of Medicine. For two-photon imaging, we used male mice to avoid potential variability due to estrus cycles. All animals used in this study were allowed free access to food and water and housed under a 12 h light/dark cycle. We used C57BL/6 (WT) mice and a bi-genic mouse (C57BL/6 genetic background) that harbored PLP-tTA (RBRC05446, RIKEN BRC, Wako, Japan) and tetO-GCaMP6 transgenes (RBRC09552, RIKEN BRC, Wako, Japan), which resulted in the expression of a fluorescence Ca^2+^ indicator, GCaMP6, in OCs/OPCs (PLP-GCaMP6 mouse) (Inamura et al., 2012; Ohkura et al., 2012; Tanaka et al., 2012). The genotype of the PLP-GCaMP6 mouse was determined using PCR with the following primer sequences: PLP-tTA, 5’-TTTCC CATGG TCTCC CTTGA GCTT-3′, 5’-CGGAG TTGAT CACCT TGGAC TTGT-3′, 5’-CTAGG CCACA GAATT GAAAG ATCT-3′, and 5′-GTAGG TGGAA ATTCT AGCAT CATCC-3′; tetO-GCaMP6, 5’-ATTTC TGAAT GGCCC AGGTC TGAG-3′, 5’-CTGCT CTGGT GTCTG TGTTA CCTG-3′, and 5′- AAGGC AGGAT GATGA CCAGG ATGT-3′. We also used tri-genic mice (C57BL/6 genetic background) harboring App^NL-G-F/NL-G-F^, PLP-tTA, and tetO-GCaMP6 transgenes. App^NL-G-F/NL-G-F^ knock-in mice express Swedish (KM670/671NL), Beyreuther/Iberian (I716F), and Arctic (E693G) mutations in the App gene because of the presence of an endogenous promoter of C57BL/6 J background (Saito et al., 2014). Each experiment was performed using mice of the appropriate age for the experiment (WT mice, 6–24 weeks old; PLP-GCaMP6 mice, 6–24 weeks old; App^NL-G-F/NL-G-F^, 16–24 weeks old; and mice crossed with App^NL-G-F/NL-G-F^ and PLP-GCaMP6 mice, 16–24 weeks old).

### 2.2. Surgery and adeno associated virus injection

Under anesthesia with ketamine (74 mg/kg, i.p.) and xylazine (10 mg/kg, i.p.), the skin was disinfected with 70% (w/v) ethanol, the skull was exposed and cleaned, and a custom-made metallic plate was firmly attached to the skull with a dental cement (C-CEM ONE; GC, Tokyo, Japan). The surface of the intact skull was coated with an acrylic-based dental resin (Super bond; Sun Medical, Shiga, Japan) to avoid drying of the surface. The metallic plate facilitated securing the mice to a manipulating frame for performing craniotomy, AAV injection, and two-photon imaging. One or two days after plate attachment, craniotomy (circular shape; 2.5 mm in diameter) and/or AAV injection were performed under isoflurane (1.0%) anesthesia. The position of the cranial window was determined by stereotaxic manipulation, according to the mouse brain atlas (centered 0.8 mm anterior and 1.2 mm lateral to the bregma). The surface of the brain was covered with 2% (w/v) agarose L (Nippon Gene, Tokyo, Japan) in saline and a glass window composed of two coverslips (2.0 mm [square] and 4.5 mm [round] in diameter; Matsunami, Osaka, Japan) joined using ultraviolet light-polymerized adhesive (NOR-61, Norland Product, Cranbury, NJ). The edge of the cranial window was sealed with an ultraviolet light-polymerized adhesive and dental cement.

### 2.3. Chemogenetic manipulation

**Figure 1:**
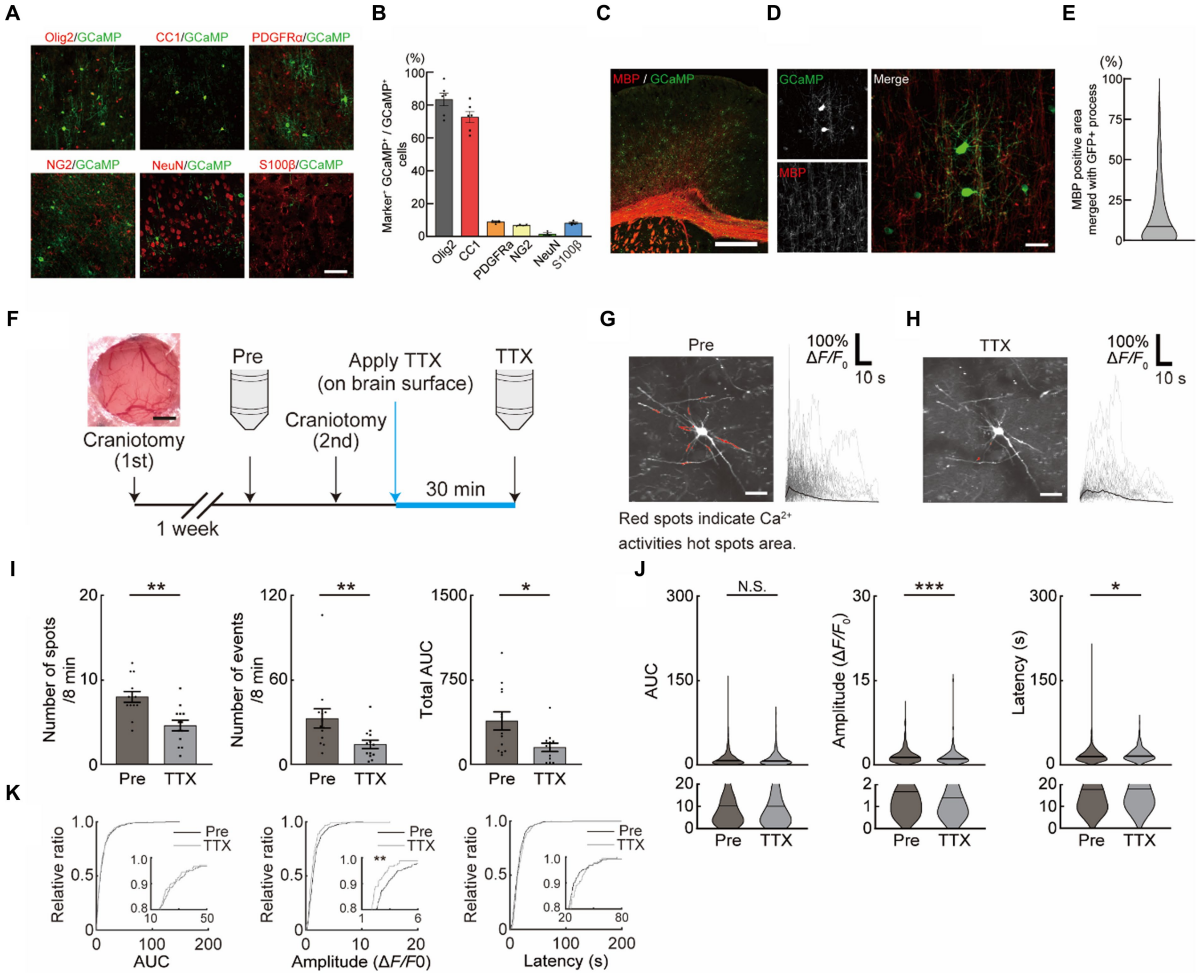
Neuronal activity-dependent Ca^2+^ activities in oligodendrocytes (OCs). **(A,B)** Representative images **(A)** and quantification **(B)** of GCaMP expression in the motor cortex of PLP-GCaMP mice and co-localization with markers for OC + oligodendrocyte precursor cell (OPC) (Olig2), OC (CC1), OPC (PDGFRα, NG2), neuron (NeuN), astrocyte (S100β) (Olig2 [83.41 ± 3.899%], CC1 [72.65 ± 3.464%], PDGFRα+ [9.979 ± 0.9322%], NG2 [6.736 ± 0.2401%], NeuN [1.481 ± 0.7246%], and S100β [8.156 ± 0.5504%]). Scale bar, 30 μm. **(C,D)** Representative images of MBP immunostaining in the motor cortex of PLP-GCaMP6 mice. Scale bars: in **(C)**, 100 μm; in **(D)**, 30 μm. **(E)** Quantitative analysis of the co-localization areas of MBP and GCaMP’ + ve processes based on immunostaining data. The proportion of MBP’ + ve processes in the processes of GCaMP’ + ve cells was about 15%. **(F)** Experimental protocol of Ca^2+^ imaging in OCs. The first craniotomy was performed one week before Pre-imaging (Pre) using two-photon microscopy. After Pre-imaging, a second craniotomy was performed and TTX was applied for 30 min, followed by a second imaging (TTX) of the same cells. **(G,H)** Representative image of GCaMP’ + ve cells of the motor cortex in PLP-GCaMP6 mice before and after TTX application. Red spots indicate the Ca^2+^ activated areas (spot). Scale bar = 30 μm. Representative Ca^2+^ traces from spots of typical GCaMP’ + ve cells are shown. **(I)** Changes in Ca^2+^ spots, Ca^2+^ events and total area under the curve (AUC) of GCaMP’ + ve cells between before and after TTX application. The number of Ca^2+^ spots and Ca^2+^ events and total AUC were significantly decreased after TTX application. Pre: *n* = 6 mice, 13 imaging fields (cells); TTX: *n* = 6 mice, 13 imaging fields (cells). *p < 0.05, **p < 0.01, Mann–Whitney *U* test. Data are presented as mean ± standard error of mean. For detailed data, check the source data file. **(J)** Changes in AUC, Amplitude, and Latency of GCaMP’ + ve cells between before and after TTX application. AUC was not significantly changed, Amplitude was significantly decreased, and Latency was significantly increased after TTX application. Pre: *n* = 6 mice, 426 events; TTX: *n* = 6 mice, 189 events. N.S., not significant, *p < 0.05, ***p < 0.001, Mann–Whitney *U* test. Violin plots show median (black line) and distribution of the data. For detailed data, check the source data file. **(K)** Proportions of AUC and Latency were not significantly changed between before and after TTX application. The proportion of lower Amplitude was significantly increased after TTX application. N.S., not significant, **p < 0.01, Kolmogorov–Smirnov test. For detailed data, check the source data file.

For chemogenetic activation of neuronal activity, a 750 nL recombinant AAV encoding the hM3D DREADD (designer receptor exclusively activated by designer drugs) vector solution was injected into the left motor cortex (M1) or the ventral-anterior/ventral-lateral thalamic nuclei (VA/VL) in the left hemisphere using a glass capillary (tip diameter, 10 μm). The axons of the VA/VL neurons extended to the ipsilateral side of M1. To visualize the Ca^2+^ response of axons, AAV1-human synapsin1 (hSyn)-axon GCaMP6s-P2A-mRuby3 (Addgene; 1 μL, 1.8 × 10^13^ viral genomes/mL) was used. The positions of M1 and VA/VL were determined by stereotaxic manipulation according to the mouse brain atlas, and a small hole was made in the skull to introduce the glass capillary into the M1 (centered 0.8 mm anterior and 1.2 mm lateral to the bregma, at a depth of 0.5 mm from the cortical surface) or VA/VL (centered 1.0 mm posterior and 1.0 mm lateral to the bregma, at a depth of 3.2 mm from the cortical surface). An AAV vector encoding the hSyn promoter driven Gq-DREAAD (a genetically modified human muscarin receptor) and AAV8-hSyn-hM3D-mCherry (Addgene; 1.5 × 10^12^ viral genomes/mL diluted in sterile saline in a 1:1 ratio) was used. After AAV injection, the small hole and the intact skull were covered with 2% (w/v) agarose gel (Nippon Gene, Tokyo, Japan) and the dental cement to avoid drying. For the combined performance of chemogenetic activation and two-photon microscopy, craniotomy above the M1 cortices was performed after AAV injection. The mice were housed individually and allowed to recover for at least 3 weeks. All experiments were started approximately 3–5 weeks after the surgical operation (AAV injection and craniotomy). Clozapine *N*-oxide (CNO, Sigma-Aldrich) was used to activate DREADD and dissolved in a 0.5 mg/mL saline stock solution. As shown in Figure 1, Supplementary Figures 2, 3, Ca^2+^ imaging of OCs was performed during a quiet resting state without CNO, and the same region was imaged again 1 to 5 h after CNO intraperitoneal injection (5 mg/kg).

### 2.4. Drug application on brain surface *in vivo*

For the *in vivo* saline or drug application on the brain surface after Pre-imaging, the cover glass was removed and saline, tetrodotoxin (5 μM TTX, Tocris Bioscience, Minneapolis, MN), 6-cyano-7-nitroquinoxaline-2,3-dione disodium (100 μM CNQX, Tocris Bioscience, Minneapolis, MN), Suramin hexasodium salt (100 μM Suramin, Tocris Bioscience, Minneapolis, MN), and pyridoxalphosphate-6-azophenyl-2′,4′-disulfonic acid tetrasodium salt (300 μM PPADS, Tocris Bioscience, Minneapolis, MN) were applied on the mice brain surface and incubated for 30 min under isoflurane (1.0%) anesthesia. After saline or drug application, the brain surface was covered with a custom-made cover window comprising two cover slips. The edge of the window was sealed with the ultraviolet light-polymerized adhesive and the dental cement.

### 2.5. Two-photon imaging

Two-photon images were acquired from the left M1/M2 cortices using a laser scanning system (C2 plus and A1, Nikon, Japan) equipped with two types of water-immersion objective lens (25×, numerical aperture [N.A.] = 1.10 and 16×, N.A. = 0.80; Nikon, Japan). The two-photon imaging based on C2 plus excitation light beams used aTi:sapphire laser (Coherent, Santa Clara, CA) and imaging based on A1 excitation light beams used aTi:sapphire laser (Spectra-Physics, Santa Clara, CA) operating at a 920–950 nm wavelength. The imaging fields were 203 × 203 μm (objective lens 25×, digital zoom 2.5) and 198 × 198 μm (objective lens 16×, digital zoom 4.0) at a 100–250 μm depth below the brain surface. The scan speed was 500 or 1,000 ms/frame. Continuous 500- or 1,000-frame serial images were acquired for each imaging field with no interval time.

### 2.6. Imaging analysis

Images were analyzed using ImageJ (National Institute of Health) and MATLAB software packages (Math Works, Natick, MA). Videos and 3D images were corrected for focal plane displacement using ImageJ plugin TurboReg and StackReg. The observed cell body area and the number of primary processes of GCaMP’ + ve (PLP’ + ve) cells were as follows and did not differ between groups (cell body area [μm^2^]: PLP-GCaMP6 mice, 86.60 ± 1.94 [8–12 weeks old], 90.68 ± 4.65 [4 months old], 88.17 ± 3.11 [5 months old], mice crossed with App^NL-G-F/NL-G-F^ and PLP-GCaMP6 mice, 88.58 ± 3.70 [4 months old], 86.91 ± 1.60 [5 months old]; number of primary processes: PLP-GCaMP6 mice, 7.80 ± 0.17 [8–12 weeks old], 7.83 ± 0.27 [4 months old], 8.04 ± 0.28 [5 months old], mice crossed with App^NL-G-F/NL-G-F^ and PLP-GCaMP6 mice, 8.00 ± 0.38 [4 months old], 7.81 ± 0.16 [5 months old], Kruskal–Wallis test followed by Dunn’s test, Supplementary Figure 1A). To estimate the M1 OC Ca^2+^ activity, the regions of interest in M1 were determined using non-negative matrix factorization. For the detection and analysis of Ca^2+^ transients, baseline fluorescence was defined as the 35th percentile of the total fluorescence intensity histogram, which was obtained during all imaging periods (F_0_). Ca^2+^ transients were calculated using the equation ΔF/F_0_ (ΔF = F-F_0_), where ΔF is the instantaneous fluorescence signal and ΔF exceeded 4 standard deviations (SDs) of the baseline fluorescence (F_0_). We used an F_0_ set at the 35th percentile of the total fluorescence distribution while re-analyzing the results. The frequency of occurrence of Ca^2+^ transients was calculated as the ratio of the total number of transients over all the imaging periods. The intensity of each Ca^2+^ transient (ΔF/F_0_) was subsequently computed using area under the curve (AUC), which was calculated by integrating area between traces representing Ca^2+^ transients and a horizontal line expressing baseline fluorescence. Amplitude was calculated as the maximum ΔF/F_0_ of each Ca^2+^ transient. Latency was calculated as the duration between the occurrence of the first Ca^2+^ transient that exceeded and was less than 4 SDs of baseline fluorescence.

### 2.7. Electrophysiology

Sixteen-channel silicone probes with recording sites measuring 177 μm^2^ (NeuroNexus Technologies), spaced 25 μm apart at depths of 3.2 mm below the cortical surface, were utilized to record neuronal activity in VA/VL neurons in 9-week-old mice under 0.5% isoflurane anesthesia. *In vivo* recordings were conducted using the Omniplex system (Plexon, Dallas, TX) at baseline, during chemogenetic activation before, and after administration of CNQX (100 μM), Suramin (100 μM) and PPADS (300 μM). Spike signals were filtered within the bandpass of 300 Hz to 8 kHz. Spikes were detected through threshold-level crossing, typically set at 50 μV (Kato et al., 2020, 2023). Single unit sorting was performed using principal component analysis in an offline sorter (Plexon).

### 2.8. Immunohistochemistry

The mice were deeply anesthetized with isoflurane and transcardially perfused with 4% paraformaldehyde in phosphate buffer (pH 7.4). Their brains were post-fixed in the same fixative overnight at 4°C, which were then extracted from the skull and equilibrated in 30% sucrose solution in phosphate buffer saline (PBS). The brains were cut in 30 μm-thick sections using a microtome (Leica Microsystems, Wetzlar, Germany). After blocking and permeabilization for 1 h in 5% bovine serum albumin and 0.5% Triton X-100 in PBS, the slices were incubated at 4°C overnight with a primary antibody diluted in PBS. After washing with PBS, the slices were subsequently incubated with a secondary antibody in PBS at room temperature for 3 h and mounted on glass slides in Fluoromount-G (Southern Biotech, Birmingham, AL). Imaging was conducted using an FV3000 confocal microscope (Olympus) with 10× (Olympus; N.A. = 0.3) and 60× oil-immersion objectives (N.A. = 0.9). The primary antibodies used in this study were as follows: anti-Olig2 (rabbit; Millipore, AB9610; 1:1000), anti-adenomatous polyposis coli (clone CC1) (mouse; Calbiochem, OP80; 1:500), anti-PDGFRα (goat; R&D systems, AF1062; 1:200), anti-NG2 (rabbit; Millipore, AB5320; 1:500), anti-NeuN (mouse; Millipore, MAB377; 1:500), anti-S100β (rabbit; Abcam, ab52642; 1:1000), anti-MBP (mouse; BioLegend, clone SMI 99; 1:100), and anti-GFP (chicken; Novus biologicals, NB100-1614; 1:2000). Amyloid β (Aβ) deposition was visualized by intraperitoneal administration of Methoxy-X04 (Tocris Bioscience, 4,920; 2 mg/kg). To visualize dying or dead cells in the brain, Fluoro-Jade C staining (Biosensis, TR-100-FJT) was performed using brain tissue from 5-month-old App^NL-G-F/NL-G-F^ and age-matched control mice, according to the manufacturer’s recommended protocol.

### 2.9. Y-maze test

The mice were housed individually before transferring to the behavioral laboratory, where they were kept during the behavioral analysis. The laboratory had a 12 h light/dark cycle (lights on at 06:00 am) and was air-conditioned and maintained at a temperature of approximately 22–23°C and a humidity of approximately 50–55%. All experiments were conducted in the light phase (06:00–18:00) and started at the same time. The Y-maze apparatus was composed of white plastic and comprised three compartments (6 cm in width, 40 cm in length, and 12 cm in height) radiating out from the center platform (6 × 6 × 6 cm triangle). In this test, each mouse was placed in the center of the maze facing toward one of the arms and was then allowed to explore the maze freely for 8 min. An arm entry was defined as the entry of four legs in an arm, and the investigator counted the sequence of entries on a television monitor from behind a partition. An alternation was defined as entries in all three arms on consecutive choices (the maximum number of alternations was the total number of entries minus 2). The percent alternation was calculated as (actual alternations divided by maximum alternations) × 100, which was the spontaneous alternation behavior of the mouse, and was considered a measure of memory performance.

### 2.10. Data analysis and statistics

Data were analyzed using GraphPad Prism 9 statistical software (GraphPad Software Inc., La Jolla, CA). All data are presented as mean ± standard error of mean. Unpaired *t*-test, Mann–Whitney *U*-, Kolmogorov–Smirnov test and Kruskal–Wallis test and Friedman test followed by Dunn’s test were used to test for statistical significance.

## 3. Results

### 3.1. Neuronal activity inhibition reduced the functional response of OCs

Neuronal activity-dependent myelination has been demonstrated in humans and rodents. Its impairment has been shown to result in asynchronized activity in late stages of motor learning tasks reducing the motor learning efficacy (Kato et al., 2020), suggesting that the OC response is associated with neuronal activity. We first assessed the functional response of OCs, which may contribute to the activity-dependent process. The mice specifically expressing the Ca^2+^ indicator (GCaMP6) under the PLP promotor (PLP-GCaMP mice) were used to observe the functional response of OCs in M1 *in vivo*. Immunohistochemical staining was performed to identify the differentiation level of GCaMP’ + ve (PLP’ + ve) cells in PLP-GCaMP mice. Approximately 85% of the GCaMP’ + ve cells were OCs/OPCs and about 75% of them were CC1 positive, which indicated that most of the GCaMP’ + ve cells were mature OCs (Figures 1A,B). In addition, the proportion of MBP’ + ve processes in the GCaMP’ + ve cells was about 15% (Figures 1C–E), suggesting that the GCaMP’ + ve cells were mature but pre-myelinating OCs. Furthermore, we only chose OC that have more than 5 processes (Supplementary Figure 1A). We performed Pre-imaging in mice that had undergone craniotomy (1st) several weeks prior to drug administration. We then performed a craniotomy (2nd), applied TTX to the brain surface to inhibit neuronal activity, and obtained the second image after 30 min (Figure 1F). Next, we observed the Ca^2+^ response of OCs *in vivo* in M1 using a two-photon microscope (Figures 1G,H and Supplementary movie 1). We analyzed the Ca^2+^ response of OCs using a MATLAB-based script (Maruyama et al., 2014). We counted the number of Ca^2+^ spots in the imaging frame (Ca^2+^ spots), number of Ca^2+^ events in the imaging frame (Ca^2+^ events), total AUC of all Ca^2+^ responses in the imaging frame (total AUC), individual AUC of all Ca^2+^ responses (AUC), individual amplitude of all Ca^2+^ responses (Amplitude), and latency of all Ca^2+^ responses (Latency) in the imaging frame. Before the application of drugs, to exclude the effects of craniotomy, we first applied saline (for 30 min) with craniotomy after Pre-imaging (initial imaging) (Supplementary Figure 1B). Saline application with craniotomy did not affect Ca^2+^ spots and Ca^2+^ events, total AUC, AUC, Amplitude, and Latency in OCs (Supplementary Figures 1C,D). Ca^2+^ spots (Pre: 8.000 ± 0.6405, TTX: 4.615 ± 0.6257, *p* = 0.0009), Ca^2+^ events (Pre: 32.77 ± 6.800, TTX: 15.00 ± 2.990, *p* = 0.0086), total AUC (Pre: 387.2 ± 80.60, TTX: 152.4 ± 36.77, *p* = 0.0256), and Amplitude (Pre: 1.650 ± 0.06944, TTX: 1.293 ± 0.1003, *p* = 0.0004) in OC Ca^2+^ responses were significantly reduced with TTX application on the brain surface (Figures 1I,J). Furthermore, applying the same analysis as described above at the individual mouse level showed that Amplitude did not change, but Ca^2+^ spots, Ca^2+^ events and total AUC significantly reduced with TTX application (Supplementary Figures 1E,F), suggesting that these factors were neuronal activity-dependent. Accumulation curves showed that number of lower amplitude Ca^2+^ activities was increased after TTX application (Figure 1K).

### 3.2. Neuronal activity promotes functional response of OCs

**Figure 2:**
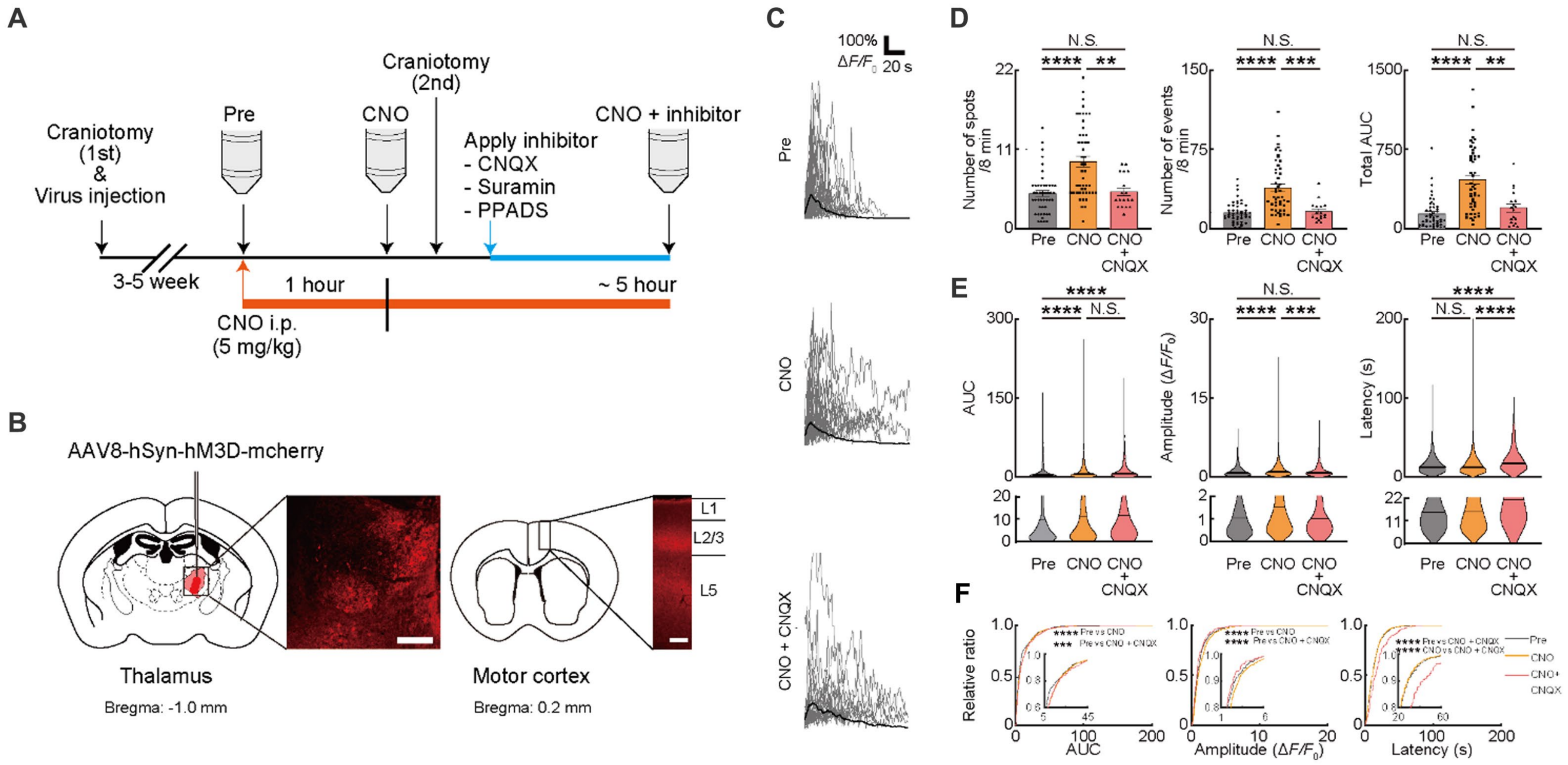
Ca^2+^ activities in oligodendrocytes (OCs) are regulated by glutamate. **(A)** Experimental protocol of the chemogenetic activation of the thalamocortical circuit and two-photon Ca^2+^ imaging with the application of neurotransmitter receptor antagonists, 6-cyano-7-nitroquinoxaline-2,3-dione disodium (CNQX), Suramin hexasodium salt (Suramin), and pyridoxalphosphate-6-azophenyl-2′,4′-disulfonic acid tetrasodium salt (PPADS). An adeno associated virus (AAV) vector coding Gq-DREAAD (hM3D) was injected into the motor thalamus and craniotomy (first) was performed. Then, 3–5 weeks after surgical operation (AAV injection and craniotomy [first]), two-photon Ca^2+^ imaging of OC was performed. A synthetic ligand clozapine *N*-oxide (CNO) was administered for hM3D activation (5 mg/kg, i.p.) after Pre-imaging (Pre). One hour after CNO application, two-photon Ca^2+^ imaging was performed again (CNO). After second imaging (CNO), neurotransmitter receptor antagonists were applied on the brain surface by craniotomy (second), and then, third imaging was performed (CNO + inhibitor). Two-photon Ca^2+^ imaging of OCs was obtained from the same cells in all imaging sessions. **(B)** Representative images showing the hM3D-mCherry expression in the motor thalamus and thalamocortical axons of motor cortices 5 weeks after the AAV injection. **(C)** Representative Ca^2+^ traces from spots of typical GCaMP’ + ve cells at Pre-imaging, and after CNO and CNO + CNQX application. **(D)** Ca^2+^ spots, Ca^2+^ events and total area under the curve (AUC) were significantly increased after CNO application. CNQX application significantly decreased these parameters. Pre: *n* = 23 mice, 48 imaging fields (cells); CNO: 23 mice, 48 imaging fields (cells); CNO + CNQX: *n* = 7 mice, 18 imaging fields (cells), N.S., not significant, **p < 0.01, ***p < 0.001, ****p < 0.0001, Kruskal–Wallis test followed by Dunn’s test. Data are presented as mean ± standard error of mean. For detailed data, check the source data file. **(E)** No statistically significant differences were detected in Latency between pre-imaging and after CNO application. AUC and Amplitude was significantly increased after CNO application. CNQX, application significantly decreased Amplitude. AUC was not significantly different between CNO and CNO + CNQX applications. Amplitude and Latency was significantly increased between CNO and CNO + CNQX applications. Pre: *n* = 23 mice, 736 events; CNO: *n* = 23 mice, 2047 events; CNO + CNQX: *n* = 7 mice, 286 events. N.S., not significant, ***p < 0.001, ****p < 0.0001, Kruskal–Wallis test followed by Dunn’s test. Violin plots show median (black line) and distribution of the data. For detailed data, check the source data file. **(F)** Proportions of larger AUC and higher Amplitude were significantly higher after CNO application than before. The proportion of lower Amplitude was significantly higher after CNO + CNQX application than after CNO application. The proportion of longer Latency was increased after CNO + CNQX application than after CNO application. Pre: *n* = 23 mice, 736 events; CNO: *n* = 23 mice, 2047 events; CNO + CNQX: *n* = 7 mice, 286 events. ***p < 0.001, ****p < 0.0001, Kolmogorov–Smirnov test. For detailed data, check the source data file.

We next promoted neuronal activity using the chemogenetic method. The AAV coding hM3Dq designer receptor, a modified human M3 muscarinic receptor under the hSyn promotor, was injected in VA/VL of PLP-GCaMP6 mice. To verify whether axonal activity increased with chemogenetic activation, we visualized the axonal Ca^2+^ responses (visualized by AAV [AAV1-hSyn-axon-GCaMP6s-P2A-mRuby3] injection into VA/VL) that projected from VA/VL neurons to M1 (Supplementary Figures 2A–E). The AUC and total AUC of axonal Ca^2+^ responses significantly increased with CNO injection and that persisted for 5 h (Supplementary Figures 2A–E). We then examined these effects on OCs in M1 (Supplementary Figure 2F). Ca^2+^ responses of OCs in M1 were visualized in PLP-GCaMP mice to quantify the changes associated with the chemogenetic activation of VA/VL axons. Ca^2+^ spots, Ca^2+^ events, total AUC, and Amplitude of Ca^2+^ responses of OCs in M1 significantly increased 1 h after CNO administration and persisted for at least 5 h (Supplementary Figures 2F–J), suggesting only a small effect of different time courses of Ca^2+^ imaging. We further assessed whether Ca^2+^ responses of OCs in M1 were affected only by VA/VL axonal activity or even with neuronal activity in M1. We injected AAV coding hM3Dq under hSyn promoter in M1 to promote activation of cortical neurons and tested whether that activated OCs in L1 of M1 (Supplementary Figure 2K). Ca^2+^ spots, Ca^2+^ events, total AUC, AUC, Amplitude, and Latency of Ca^2+^ responses of OCs in L1 of M1 did not show any detectable changes, suggesting the minimum impact of neurons of M1 but significant impact of VA/VL axonal activity on OCs in L1 of M1 (Supplementary Figures 2L–O). These results indicated that the increased Ca^2+^ response in OC requires enhanced activity of VA/VL axons projecting to M1 (Figures 2A,B). We then measured the Ca^2+^ response in OCs with chemogenetic activation of axons projecting from VA/VL neurons. Ca^2+^ spots (Pre: 4.885 ± 0.4102, CNO: 9.333 ± 0.7324, *p* < 0.0001), Ca^2+^ events (Pre: 15.22 ± 1.449, CNO: 38.72 ± 3.534, *p* < 0.0001), total AUC (Pre: 142.4 ± 20.83, CNO: 465.1 ± 44.16, *p* < 0.0001), AUC (Pre: 9.249 ± 0.5549, CNO: 11.48 ± 0.3921, *p* < 0.0001) and Amplitude (Pre: 1.141 ± 0.04281, CNO: 1.422 ± 0.03225, *p* < 0.0001) were increased after CNO administration, consistent with TTX application, suggesting that these factors were affected by neuronal transmitters such as glutamate and ATP. OCs express receptors for ATP and glutamate such as AMPAR, P2X-R, and P2Y-R (Groc et al., 2002; Agresti et al., 2005; Fields and Burnstock, 2006; Zonouzi et al., 2011; Fannon et al., 2015; Feng et al., 2015; Gautier et al., 2015; Spitzer et al., 2019). We, therefore, attempted to identify specific transmitters affecting these factors of OC Ca^2+^ responses by treating with specific inhibitors. Using *in vivo* electrophysiology, we first tested whether neuronal activity in VA/VL was altered by CNQX, Suramin, PPADS application on the brain surface during chemogenetic activation. We observed no significant changes in the activity of VA/VL neurons activated by chemogenetic method before and after administration of these inhibitors (Supplementary Figures 2P–R). We next attempted to evaluate the contribution of glutamatergic transmission in the activity-dependent OC responses. To inhibit AMPAR expressed on the cell surface of OCs, the antagonist for AMPAR (CNQX) was used after CNO injection (Figure 2A). CNQX applied after the second craniotomy showed minimum effects on Ca^2+^ responses of OCs (Supplementary Figures 1A–C). CNQX treatment significantly reduced Ca^2+^ spots (CNO: 9.333 ± 0.7324, CNO + CNQX; 5.111 ± 0.5417, *p* = 0.0032), Ca^2+^ events (CNO: 38.72 ± 3.534, CNO + CNQX: 16.24 ± 2.316, *p* = 0.0002), total AUC (CNO: 465.1 ± 44.16, CNO + CNQX: 197.9 ± 38.29, *p* = 0.0027), Amplitude (CNO: 1.141 ± 0.04281, CNO + CNQX: 1.124 ± 0.06736, *p* = 0.0004) and Latency (CNO: 15.46 ± 0.2839, CNO + CNQX: 21.31 ± 0.9205, *p* < 0.0001) of OCs, indicating that AMPAR mediating Ca^2+^ activity contribute to of OC responses (consistent with Figure 1) (Figures 2C–F). The accumulation curve showed that the number of higher Amplitude Ca^2+^ activities was increased after CNO administration. On the other hand, the number of lower Amplitude Ca^2+^ activities was increased after CNQX application (Figure 2F).

**Figure 3:**
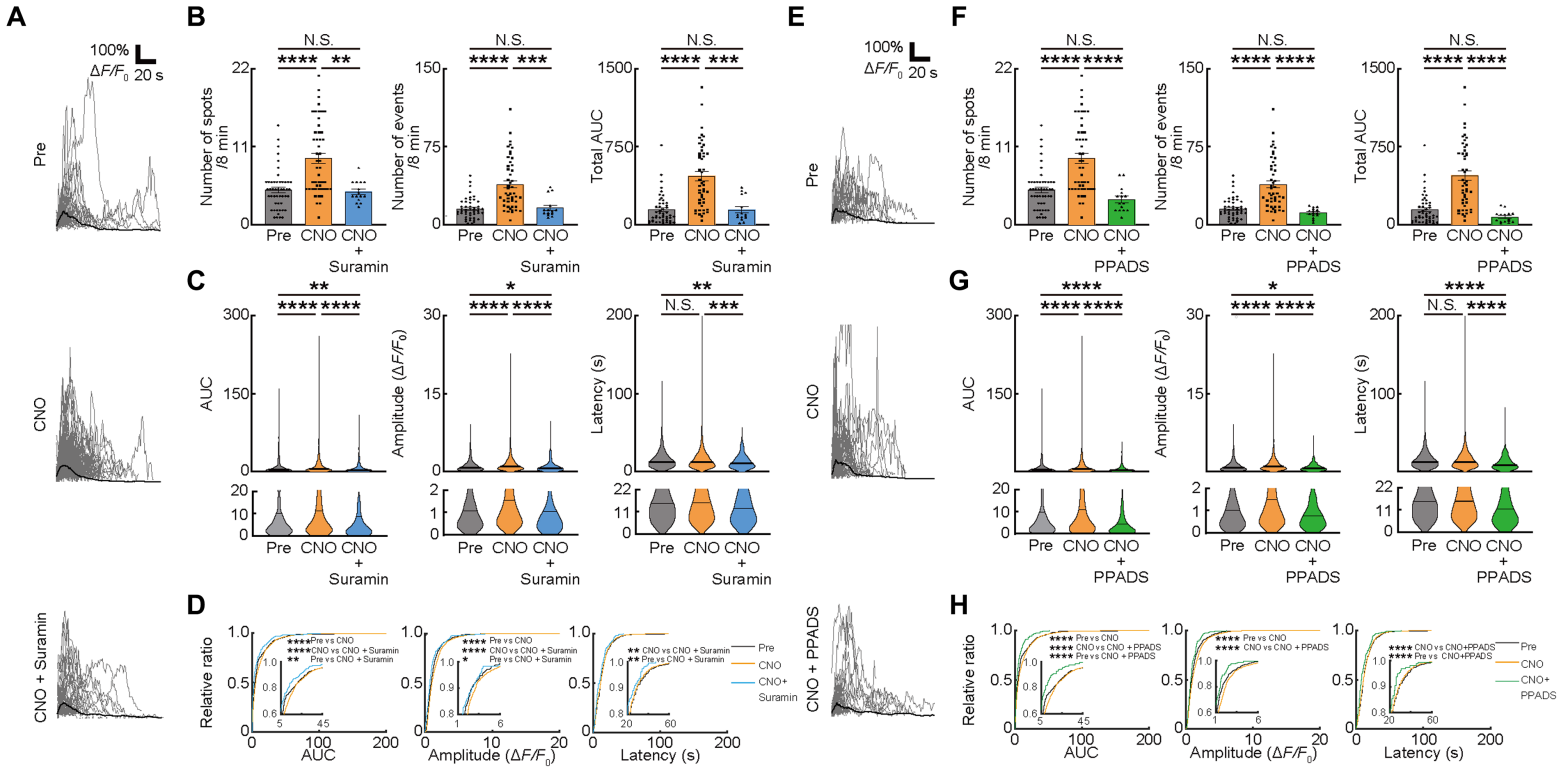
Ca^2+^ activities in oligodendrocytes (OCs) are regulated by ATP. **(A,E)** Representative Ca^2+^ traces from spots of typical GCaMP’ + ve cells at Pre-imaging, and after CNO, CNO + Suramin, and CNO + PPADS applications. **(B,F)** Ca^2+^ spots, Ca^2+^ events and total area under the curve (AUC) were significantly increased after CNO application. Suramin, and PPADS applications significantly decreased these parameters. Pre: *n* = 23 mice, 48 imaging fields (cells); CNO: 23 mice, 48 imaging fields (cells); CNO + Suramin: 9 mice, 15 imaging fields (cells): CNO + PPADS: *n* = 7 mice, 15 imaging fields (cells), N.S., not significant, **p < 0.01, ***p < 0.001, ****p < 0.0001, Kruskal–Wallis test followed by Dunn’s test. Data are presented as mean ± standard error of mean. For detailed data, check the source data file. **(C,G)** No statistically significant differences were detected in Latency between pre-imaging and after CNO application. AUC and Amplitude was significantly increased after CNO application. Suramin, and PPADS applications significantly decreased Amplitude. Suramin and PPADS significantly decreased AUC, Amplitude and Latency. Pre: *n* = 23 mice, 736 events; CNO: *n* = 23 mice, 2047 events; CNO + Suramin: *n* = 9 mice, 289 events; CNO + PPADS: *n* = 7 mice, 289 events. N.S., not significant, *p < 0.05, **p < 0.01, ***p < 0.001, ****p < 0.0001, Kruskal–Wallis test followed by Dunn’s test. Violin plots show median (black line) and distribution of the data. For detailed data, check the source data file. **(D,H)** Proportions of larger AUC and higher Amplitude were significantly higher after CNO application than before. The proportion of lower Amplitude was significantly higher after CNO + Suramin, and CNO + PPADS applications than after CNO application. The number of shorter Latency Ca^2+^ activities was increased after CNO + Suramin and Suramin + PPADS applications than after CNO application. Pre: *n* = 23 mice, 736 events; CNO: *n* = 23 mice, 2047 events; CNO + Suramin: *n* = 9 mice, 289 events; CNO + PPADS: *n* = 7 mice, 289 events. *p < 0.05, **p < 0.01, ****p < 0.0001, Kolmogorov–Smirnov test. For detailed data, check the source data file.

We further examined whether these factors changed with the inhibition of ATP signaling. To inhibit P2 receptors, the antagonist for P2 receptors (Suramin) and P2X and Y receptors (PPADS) were used after CNO injection (Figure 2A). Inhibition of P2 receptors with Suramin reduced Ca^2+^ spots (CNO: 9.333 ± 0.7324, CNO + Suramin; 4.633 ± 0.3887, *p* < 0.00223), Ca^2+^ events (CNO: 38.72 ± 3.534, CNO + Suramin: 16.03 ± 2.476, *p* = 0.0004), total AUC (CNO: 465.1 ± 44.16, CNO + Suramin: 141.2 ± 30.56, *p* = 0.0001), AUC (CNO: 11.48 ± 0.3921, CNO + Suramin: 7.373 ± 0.7401, *p* < 0.0001), Amplitude (CNO: 1.422 ± 0.03225, CNO + Suramin: 1.021 ± 0.07104, *p* < 0.0001) and Latency (CNO: 15.46 ± 0.2839, CNO + Suramin: 12.70 ± 0.5211, *p* < 0.0001) of OCs (Figures 3A–D). Consistent with data from Suramin application, inhibition of P2X and Y receptors using PPADS reduced Ca^2+^ spots (CNO: 9.333 ± 0.7324, CNO + PPADS; 3.533 ± 0.4641, *p* < 0.0001), Ca^2+^ events (CNO: 38.72 ± 3.534, CNO + PPADS: 11.23 ± 1.218, *p* < 0.0001), total AUC (CNO: 465.1 ± 44.16, CNO + PPADS: 72.35 ± 14.37, *p* < 0.0001), AUC (CNO: 11.48 ± 0.3921, CNO + PPADS: 5.320 ± 0.5848, *p* < 0.0001), Amplitude (CNO: 1.422 ± 0.03225, CNO + PPADS: 0.8632 ± 0.05715, *p* < 0.0001) and Latency (CNO: 15.46 ± 0.2839, CNO + PPADS: 12.67 ± 0.7579, *p* < 0.0001) of OCs (Figures 3E–H). The accumulation curve showed that the number of lower Amplitude Ca^2+^ activities was increased after Suramin and PPADS application (Figures 3D,H). As previously described, the chemogenetic activation of VA/VL neurons promoted activity in VA/VL axons and OCs in L1 of M1 for 5 h. To verify that the effect of antagonists was not due to differences in the time course after chemogenetic activation, we performed Pre-imaging followed by CNO administration. Subsequently, we applied saline or PPADS following craniotomy, and finally performed imaging 1 h after CNO administration with saline or PPADS (imaging 1 h after CNO + saline and 1 h after CNO + PPADS, respectively). The results showed that Ca^2+^ responses in OCs persisted in the saline group, whereas in the PPADS group, Ca^2+^ responses in OCs significantly reduced, indicating an effect of neurotransmitter antagonists (Supplementary Figures 3A,B). In addition, glutamate and ATP differentially affected the latency of Ca^2+^ transients in OCs (Figures 2E,F, 3C,D,G,H). Suramin and PPADS application significantly shortened the latency of Ca^2+^ transients in OCs, suggesting that ATP induced longer latency of Ca^2+^ transients. In contrast, CNQX lengthened the latency of Ca^2+^ transients in OCs, indicating that glutamate induced faster latency of Ca^2+^ transients in OCs. Thus, the latency of Ca^2+^ responses in OCs is regulated by different types of neurotransmitters previously known to elicit Ca^2+^ responses in OCs (Gallo et al., 1996; Bergles et al., 2000; Chittajallu et al., 2004; Lin and Bergles, 2004; Lin et al., 2005; Kukley et al., 2007; Káradóttir et al., 2008; Kougioumtzidou et al., 2017). Furthermore, our analysis of Ca^2+^ responses in OCs at the individual mouse level was similar to that at the imaging fields (cells) level (Supplementary Figures 3C–H).

### 3.3. OC responses in mice model of AD

**Figure 4:**
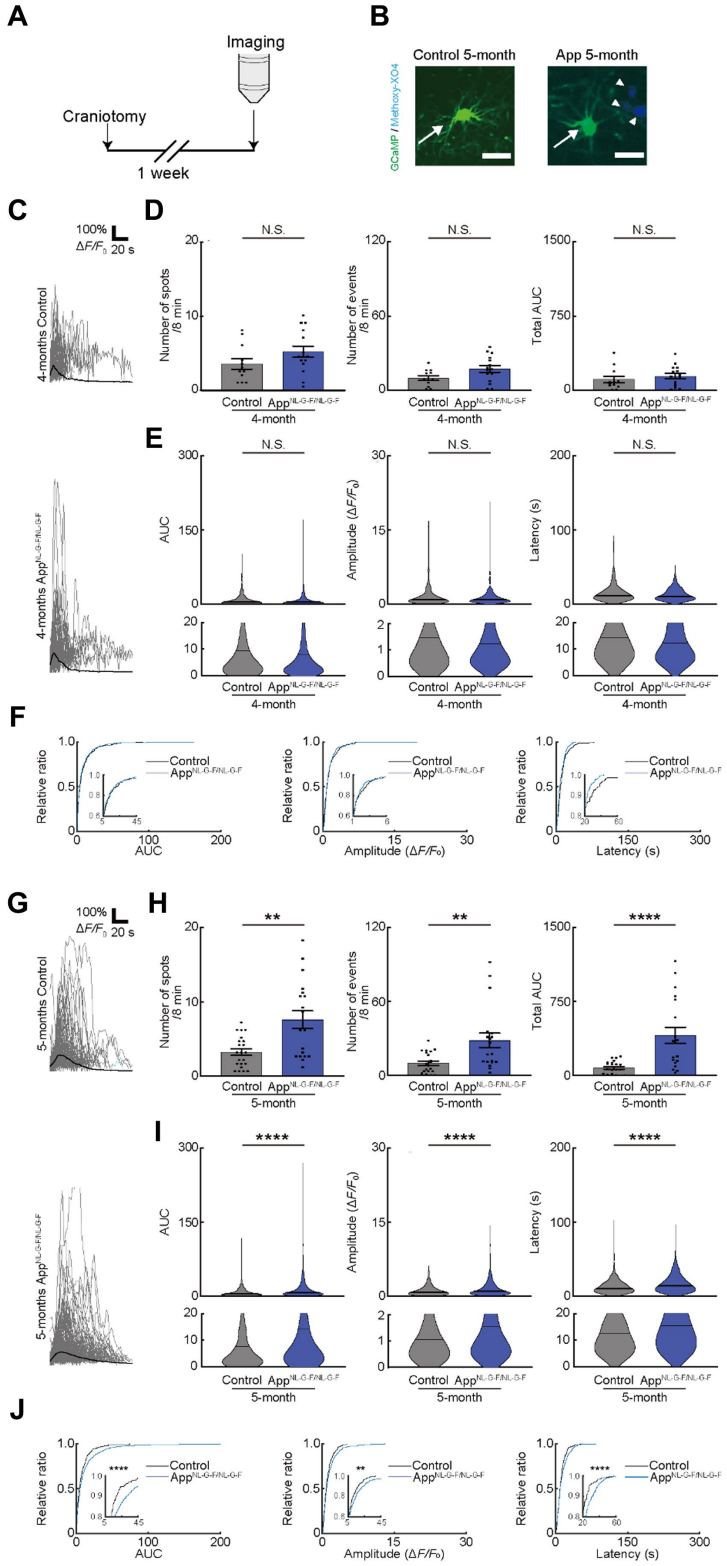
Ca^2+^ activity of oligodendrocytes (OCs) is altered in 5-month-old App^NL-G-F/NL-G-F^ mice. **(A)** Experimental protocol of two-photon Ca^2+^ imaging of OCs in App^NL-G-F/NL-G-F^ mice. For this purpose, transgenic mice (PLP-GCaMP6) were crossed with App^NL-G-F/NL-G-F^ mice and craniotomy was performed one week before imaging. **(B)** Representative image of GCaMP’ + ve cells in the motor cortex of 5-month-old age-matched control and App^NL-G-F/NL-G-F^ mice. Arrow indicates a GFP^+^ cell. Arrowhead indicates amyloid β deposition visualized by intraperitoneal administration of Methoxy-X04. Scale bar, 30 μm. **(C)** Representative Ca^2+^ traces from typical GCaMP’ + ve cell processes from 4-month-old age-matched control and AppNL-G-F/NL-G-F mice. **(D)** No statistically significant differences were detected in Ca^2+^ spots, Ca^2+^ events and total area under the curve (AUC) between Control and App^NL-G-F/NL-G-F^ mice at 4 months of age. Control: *n* = 6 mice, 12 imaging fields (cells); App^NL-G-F/NL-G-F^: *n* = 6 mice, 15 imaging fields (cells). N.S., not significant by Mann–Whitney *U*-test. Data are presented as mean ± standard error of mean. For detailed data, check the source data file. **(E)** No statistically significant differences were detected in the AUC, Amplitude, and Latency between Control and App^NL-G-F/NL-G-F^ mice at 4 months of age. Control; *n* = 6 mice, 233 events; App^NL-G-F/NL-G-F^; *n* = 6 mice, 418 events. N.S., not significant, Mann–Whitney *U*-test. Violin plots show median (black line) and distribution of the data. For detailed data, check the source data file. **(F)** Proportions of larger AUC, Amplitude, and Latency were not significantly different between Control and App^NL-G-F/NL-G-F^ mice at 4 months of age. Control; *n* = 6 mice, 233 events; App^NL-G-F/NL-G-F^; *n* = 6 mice, 418 events. N.S., not significant, Kolmogorov–Smirnov test. For detailed data, check the source data file. **(G)** Representative Ca^2+^ traces from typical GCaMP’ + ve cell processes from 5-month-old age-matched control and App^NL-G-F/NL-G-F^ mice. **(H)** Ca^2+^ spots, Ca^2+^ events and total AUC were significantly higher in App^NL-G-F/NL-G-F^ mice than in Control mice at 5 months of age. Control: *n* = 8 mice, 22 imaging fields (cells); App^NL-G-F/NL-G-F^: *n* = 8 mice, 18 imaging fields (cells). **p < 0.01, ****p < 0.0001, Mann–Whitney *U*-test. Error bar shows mean ± standard error of mean. For detailed data, check the source data file. **(I)** AUC, Amplitude, and Latency were significantly higher in App^NL-G-F/NL-G-F^ mice than in Control mice at 5 months of age. Control: *n* = 8 mice, 364 events; App^NL-G-F/NL-G-F^: *n* = 8 mice, 877 events. ****p < 0.0001, Mann–Whitney *U*-test. Violin plots show median (black line) and distribution of the data. For detailed data, check the source data file. **(J)** Proportions of larger AUC and higher Amplitude and Latency were significantly higher in App^NL-G-F/NL-G-F^ mice than in Control mice at 5 months of age. Control: *n* = 8 mice, 364 events; App^NL-G-F/NL-G-F^: *n* = 8 mice, 877 events. **p < 0.01, ****p < 0.0001, Kolmogorov–Smirnov test. For detailed data, check the source data file.

**Figure 5:**
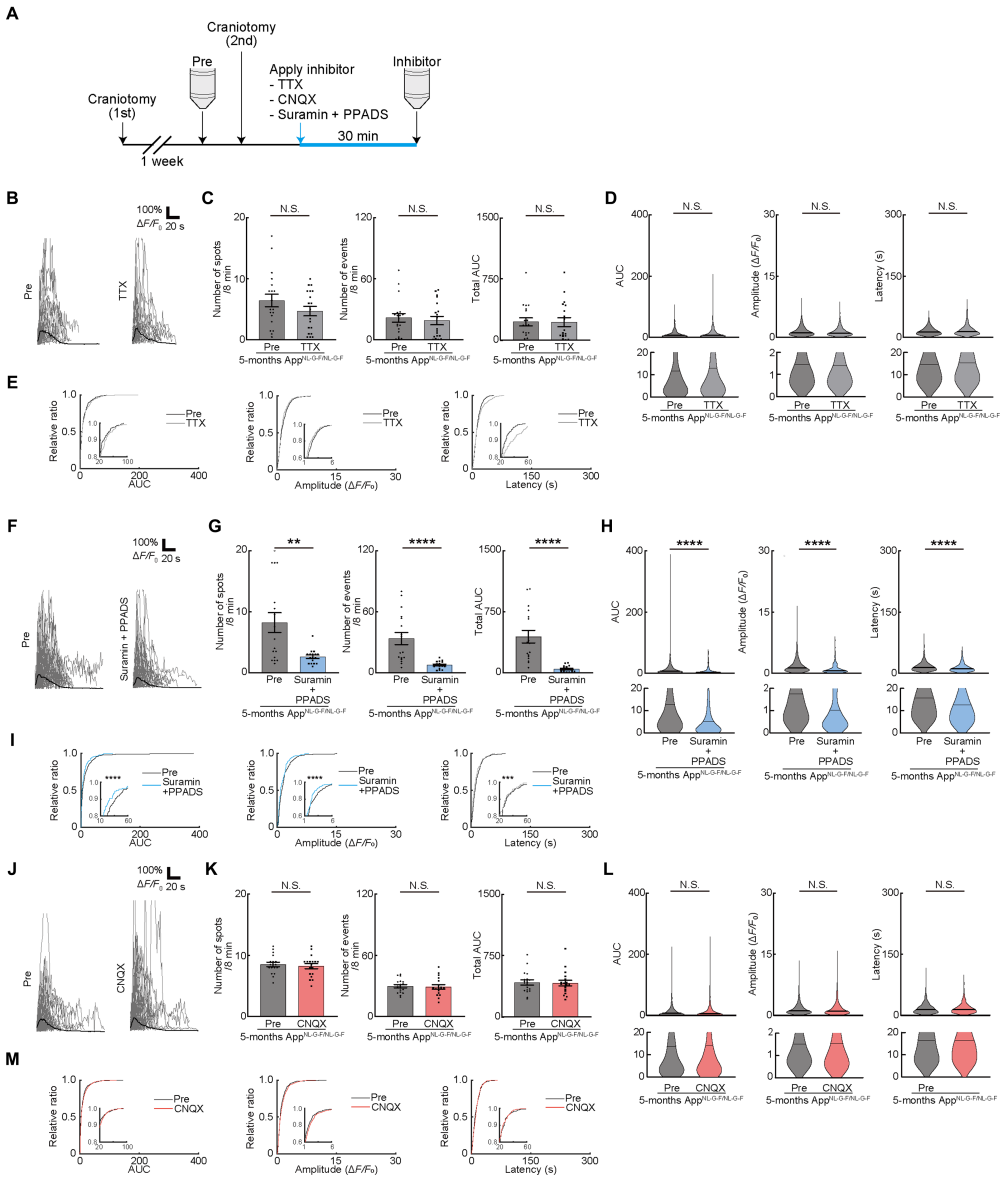
Pharmacological manipulation of oligodendrocyte (OC) Ca^2+^ activities in App^NL-G-F/NL-G-F^ mice at 5 months. **(A)** Experimental protocol of two-photon Ca^2+^ imaging of OCs in App^NL-G-F/NL-G-F^ mice at 5 months of age. One week after craniotomy (first), two-photon Ca^2+^ imaging was performed (Pre). After Pre- imaging, TTX or neurotransmitter receptor antagonists (CNQX or Suramin and PPPADS) were applied on the brain surface by craniotomy (second), and then, second imaging was performed (Inhibitor). Two-photon Ca^2+^ imaging of OCs was obtained from the same cells in all imaging sessions. **(B)** Representative Ca^2+^ traces from typical GCaMP’ + ve cell processes after TTX application. **(C)** No statistically significant differences were detected in Ca^2+^ spots, Ca^2+^ events and total area under the curve (AUC) between before and after TTX application. Pre: *n* = 9 mice, 19 imaging fields (cells); TTX: *n* = 9 mice, 19 imaging fields (cells). N.S., not significant, Mann–Whitney *U*-test. Data are presented as the mean ± standard error of mean. For detailed data, check the source data file. **(D)** No statistically significant differences were detected in AUC, Amplitude, and Latency between before and after TTX application. Pre: *n* = 9 mice, 611 events; TTX: *n* = 9 mice, 517 events. N.S., not significant, Mann–Whitney *U*-test. Violin plots show median (black line) and distribution of the data. For detailed data, check the source data file. **(E)** Proportions of AUC, Amplitude, and Latency were not significantly different between before and after TTX application. Pre: *n* = 9 mice, 611 events; TTX: *n* = 9 mice, 517 events. N.S., not significant, Kolmogorov–Smirnov test. **(F)** Representative Ca^2+^ traces from typical GCaMP’ + ve cell processes after Suramin + PPADS application. **(G)** Ca^2+^ spots, Ca^2+^ events and total AUC were significantly decreased after Suramin + PPADS application. Pre: *n* = 10 mice, 17 imaging fields (cells); Suramin + PPADS: *n* = 10 mice, 17 imaging fields (cells). **p < 0.01, ****p < 0.0001, Mann–Whitney *U*-test. Data are presented as the mean ± standard error of mean. For detailed data, check the source data file. **(H)** AUC, Amplitude, and Latency were significantly decreased after Suramin + PPADS application. Pre: *n* = 10 mice, 777 events; Suramin + PPADS: *n* = 10 mice, 208 events. ****p < 0.0001, Mann–Whitney *U*-test. Violin plots show median (black line) and distribution of the data. For detailed data, check the source data file. **(I)** Proportions of smaller AUC, lower Amplitude, and shorter Latency were significantly increased after Suramin + PPADS application. Pre: *n* = 10 mice, 777 events; Suramin + PPADS: *n* = 10 mice, 208 events. ***p < 0.001, ****p < 0.0001, Kolmogorov–Smirnov test. For detailed data, check the source data file. **(J)** Representative Ca^2+^ traces from typical GCaMP’ + ve cell processes after CNQX application. **(K)** No statistically significant differences were detected in Ca^2+^ spots, Ca^2+^ events, and total area under the curve (AUC) between before and after CNQX application. Pre: *n* = 10 mice, 18 imaging fields (cells); CNQX: *n* = 10 mice, 18 imaging fields (cells). N.S., not significant, Mann–Whitney *U*-test. Data are presented as the mean ± standard error of mean. For detailed data, check the source data file. **(L)** No statistically significant differences were detected in AUC, Amplitude, and Latency between before and after CNQX application. Pre: *n* = 10 mice, 822 events; CNQX: *n* = 10 mice, 780 events. N.S., not significant, Mann–Whitney *U*-test. Violin plots show median (black line) and distribution of the data. For detailed data, check the source data file. **(M)** Proportions of AUC, Amplitude, and Latency were not significantly different between before and after CNQX application. Pre: *n* = 10 mice, 822 events; CNQX: *n* = 10 mice, 780 events. N.S., not significant, Kolmogorov–Smirnov test. For detailed data, check the source data file.

The release of ATP from the dying and dead cells of the damaged brain (Honda et al., 2001; Davalos et al., 2005; Haynes et al., 2006; Matute et al., 2007; Duan et al., 2009) increases extracellular ATP concentrations and contributes to AD pathology (Burnstock, 2008; Cieslak and Wojtczak, 2018; Francistiova et al., 2020). Therefore, we attempted to detect the difference in OC responses in mice models of AD. We used App^NL-G-F/NL-G-F^ mice, as reported previously and provided by Dr. Saido, as the mice models of AD (Saito et al., 2014). We first assessed the behavioral abnormalities and found no detectable changes in 4- or 5-month-old App^NL-G-F/NL-G-F^ mice. However, 6-month-old App^NL-G-F/NL-G-F^ mice had impaired alteration ratio (Supplementary Figure 4A), though the number of entries was not impaired, which is consistent with the result of a previous study (Saito et al., 2014). Since white matter lesions appear in head magnetic resonance imaging of patients with AD before the onset of cognitive decline (Lee et al., 2016), we next focused on the period before the onset of behavioral abnormalities at 4 or 5 months of age, by observing OC Ca^2+^ responses in App^NL-G-F/NL-G-F^ mice (Figures 4A,B). Ca^2+^ spots, Ca^2+^ events, total AUC, AUC, Amplitude, and Latency were not increased in 4-month-old App^NL-G-F/NL-G-F^mice, suggesting that OCs in 4-month-old App^NL-G-F/NL-G-F^ mice did not show abnormal Ca^2+^ activities (Figures 4C–F). In contrast, Ca^2+^ spots, Ca^2+^ events, total AUC, AUC, Amplitude, and Latency increased significantly in 5-month-old App^NL-G-F/NL-G-F^ mice than in control mice (Supplementary movies 2, 3), both at the imaging field (cells) (Figures 4G–J; see also source data file) and individual mouse levels (Supplementary Figures 4B,C), suggesting abnormal Ca^2+^ activities are present in 5-month-old App^NL-G-F/NL-G-F^ mice. Furthermore, we confirmed by immunohistochemical staining that the differentiation levels of GCaMP’ + ve cells do not differ between 5-month-old App^NL-G-F/NL-G-F^ mice and age-matched control mice (Supplementary Figure 4D). These increased abnormal Ca^2+^ activities in OCs were not inhibited by TTX treatment (Figure 5A), suggesting that neuronal activity did not contribute to increased abnormal Ca^2+^ activities in OCs of 5-month-old App^NL-G-F/NL-G-F^ mice (Figures 5B–E). Therefore, we hypothesized that dying or dead cells in 5-month-old App^NL-G-F/NL-G-F^ mice released ATP, enhancing the functional OC responses. To test this hypothesis, we first stained 5-month-old App^NL-G-F/NL-G-F^ mice with Fluoro-Jade C to investigate whether they had more dying or dead cells compared to age-matched control mice. As expected, the number of dying or dead cells was significantly increased in 5-month-old App^NL-G-F/NL-G-F^ mice compared to that in age-matched control mice (Supplementary Figure 4E). To further explore whether the abnormal Ca^2+^ activities in OCs were due to the increased ATP in 5-month-old App^NL-G-F/NL-G-F^ mice, Suramin and PPADS were administered to 5-month-old App^NL-G-F/NL-G-F^ mice (Figure 5A). Suramin and PPADS application significantly reduced Ca^2+^ spots (Pre: 8.206 ± 1.613, Suramin + PPADS: 2.618 ± 0.2829, *p* = 0.0044), Ca^2+^ events (Pre: 33.56 ± 5.976, Suramin + PPADS: 7.559 ± 0.8920, *p* < 0.0001), total AUC (Pre: 443.3 ± 77.70, Suramin + PPADS: 46.20 ± 9.347, *p* < 0.0001), AUC (Pre: 13.86 ± 0.8542, Suramin + PPADS: 7.286 ± 0.8543, *p* < 0.0001), Amplitude (Pre: 1.751 ± 0.06353, Suramin + PPADS: 1.041 ± 0.08171, *p* < 0.0001). Suramin and PPADS application also reduced Latency (Pre: 15.71 ± 0.4135, Suramin + PPADS: 12.59 ± 0.7377, *p* < 0.0001) both at imaging fields (cells) (Figures 5F–H) and individual mouse levels (Supplementary Figures 4F,G). Accumulation curves also showed a significant change in the distribution of AUC, Amplitude and Latency after Suramin + PPADS application (Figure 5I). In contrast, CNQX application did not show detectable changes in OC Ca^2+^ responses in 5-month-old App^NL-G-F/NL-G-F^ mice (Figures 5J–M). Furthermore, properties of Ca^2+^ responses (AUC, Amplitude, and Latency) did not correlate with the distance between Ca^2+^ spots and Aβ deposition, but they were definitely more effectively suppressed by ATP inhibitors in OCs of 5-month-old App^NL-G-F/NL-G-F^ mice than in OCs of age-matched control mice (Supplementary Figures 4H,I). These data suggest that OCs in 5-month-old App^NL-G-F/NL-G-F^ mice mainly receive ATP released from dying or dead cells.

## 4. Discussion

In this research, we observed OC Ca^2+^ responses. Activity-dependent glutamate and ATP release from neurons or astrocytes trigger OC responses with different properties of Ca^2+^ responses. In mice models of AD, these activity-dependent responses were lost but higher frequency of ATP release induced Ca^2+^ responses due to neurodegeneration.

Myelination is essential for efficient information processing in the brain. OPCs originate in the ventral ventricular layer of the zona limitans intrathalamica in the mesencephalon. From here, OPCs proliferate and migrate to be widely distributed in the brain. After they settle in the brain, OPCs differentiate and myelinate the axons to regulate the conduction velocity. Previous studies showed that proliferation and differentiation of OPCs and myelination depend on neuronal activity (Menn et al., 2006; Xing et al., 2014). TTX injection into the eyes reduced OPC proliferation, and inhibition of neurotransmitter release by the botulinus or tetanus toxin resulted in deficient activity-dependent myelination due to impaired local translation of myelin basic protein (Barres and Raff, 1993). OCs express various receptors for neurotransmitters such as AMPA, NMDA (N-methyl-D-aspartic acid), gamma-aminobutyric acid B, and purinergic P1 and P2 receptors. Growing evidence suggested that neurotransmitters regulate migration and proliferation of OPCs, differentiation into OCs, and myelination in mature OCs (Nishiyama et al., 2021). Adenosine, the metabolite of ATP, has been shown to inhibit proliferation of OPCs, in contrast to ATP, which itself is a contradictory result. In addition, OPCs form a synapse-like structure with axons and receive glutamate on AMPAR, which promotes OPC proliferation and inhibits their differentiation into OCs (Agresti et al., 2005; Kato et al., 2018). AMPA, NMDA, and P1 and P2 signaling mediates Ca^2+^ transients in OCs/OPCs that regulate downstream signaling. The developmental changes in these receptor expressions have been known (Matute et al., 2007; Spitzer et al., 2019).

Here, we showed that glutamate-mediated Ca^2+^ transients in OC exhibited a short decay, but ATP-mediated Ca^2+^ transients in OC exhibited a long decay. These differences may induce various gene expressions to regulate their fate differently. Deficient activity-dependent myelin regulation in adult mice impaired temporal regulation of spike arrival, leading to increased spontaneous neuronal activity and reduced movement-induced neuronal activity in the primary motor cortex, which in turn impaired the motor learning process (Kato et al., 2020). Although a previous study demonstrated that Ca^2+^ transients in myelin sheaths of L5 or L6 PLP-positive cells in mice are not dependent on neuronal activity but are dependent on mitochondria (Battefeld et al., 2019), our study showed that L1 GCaMP’ + ve (PLP’ + ve) cells were predominantly CC1 positive but a few were MBP positive, suggesting that pre-myelinating OCs showed neuronal activity dependent responses.

ATP is known to be released abundantly from degenerative brain tissue. Released ATP activates P2X7 signaling, which promotes inflammatory response in neurodegenerative diseases, such as AD, amyotrophic lateral sclerosis, multiple sclerosis, and spinal cord injury that cause apoptosis or dysmorphic changes in OCs, and ultimately induces de-myelination to promote disease progression. Specifically, in studies in patients with AD and mice models of AD, spatial transcriptome analysis revealed that genetic changes in OCs around Aβ deposition occur early in the disease (Chen et al., 2020). Furthermore, OCs/OPCs have been found to show accelerated aging in mice models of AD and removal of these cells have been found to improve their cognitive function (Zhang et al., 2019). We used mice models of AD and found that the abundant ATP released from degenerative brain tissue increased abnormal Ca^2+^ responses in OCs, which may cause dysmorphic changes in OCs. In addition, the increased abnormal Ca^2+^ responses in OCs may trigger myelin defects, abnormal myelination, or abnormal turnover in OPC differentiation. A recent study suggested that structural defects in myelin promote Aβ deposition and is an upstream risk factor for AD (Depp et al., 2023), which may be associated with abnormal Ca^2+^ responses in OC observed at 5 months of age prior to the onset of behavioral abnormalities. Further studies are needed to clarify the effects of abnormal Ca^2+^ responses in OCs on disease progression.

## Data availability statement

The original contributions presented in the study are included in the article/Supplementary material, further inquiries can be directed to the corresponding author.

## Ethics statement

All experimental protocols used in animals were approved by the Animal Care and Use Committees of Nagoya University Graduate School of Medicine and Kobe University Graduate School of Medicine. The study was conducted in accordance with the local legislation and institutional requirements.

## Author contributions

KY, DK, and HW designed research and wrote the paper. KY, DK, and SS performed research. KY, DK, SS, IT, and HW analyzed data. All authors contributed to the article and approved the submitted version.
