# Supplementary material for: Activity-dependent oligodendrocyte calcium dynamics and their changes in Alzheimer’s disease
Source: Front Cell Neurosci. 2023 Oct 31;17:1154196. doi: 10.3389/fncel.2023.1154196 (PMC10644703; doi:10.3389/fncel.2023.1154196)
Supplement: Supplementary file 1 [file Table_1.docx]

Supplementary Material

Activity-Dependent Oligodendrocyte Calcium Dynamics and Their Changes in Alzheimer’s Disease

Kenji Yoshida, Daisuke Kato, Shouta Sugio, Ikuko Takeda, Hiroaki Wake^*^

*** Correspondence:** Hiroaki Wake: hirowake@med.nagoya-u.ac.jp

## Supplementary Figure


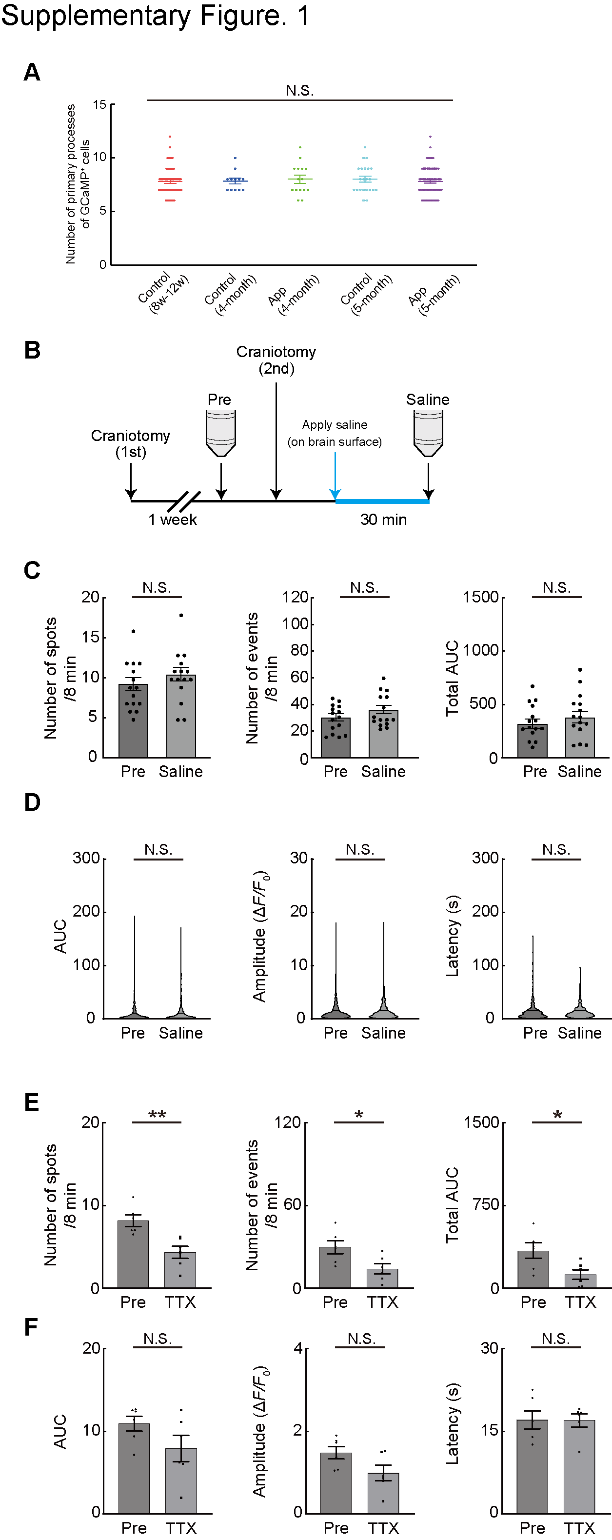


**Supplementary Figure 1.** Experimental protocol of *in vivo* Ca^2+^ imaging in oligodendrocytes (OCs) and neuronal activity-dependent Ca^2+^ responses in OCs at the individual mouse level before and after tetrodotoxin (TTX) application

(A) Plot of the number of primary processes of GCaMP’+ve cells in all experiments. For detailed data, check the source data file.

(B) Experimental protocol of Ca^2+^ imaging in OCs was designed to exclude the effects of craniotomy, rather than those of drug application. The craniotomy (first) was performed one week before Pre-imaging (Pre) using two-photon microscopy. After Pre-imaging (Pre), a second craniotomy was performed and saline was applied for 30 min, followed by a second imaging (Saline) of the same cells.

(C) There is no difference in the number of Ca^2+^ spots and Ca^2+^ events and total area under the curve (AUC) of GCaMP’+ve cells between before and after saline application. N.S., not significant, Mann Whitney U test. Data are presented as mean ± standard error of mean. For detailed data, check the source data file.

(D) There is no difference in AUC, Amplitude, and Latency of GCaMP’+ve cells between before and after saline application. N.S., not significant, Mann Whitney U test. Violin plots show median (black line) and distribution of the data. For detailed data, check the source data file.

(E) Changes in the number of Ca^2+^ spots and Ca^2+^ events and AUC of GCaMP’+ve cells between before and after TTX application at individual mouse level. **P* < 0.05, ***P* < 0.01, Mann Whitney U test. Data are presented as mean ± standard error of mean. For detailed data, check the source data file.

(F) There is no difference in AUC, Amplitude, and Latency of GCaMP’+ve cells between before and after TTX application at individual mouse level. N.S., not significant, Mann Whitney U test. Data are presented as mean ± standard error of mean. For detailed data, check the source data file.


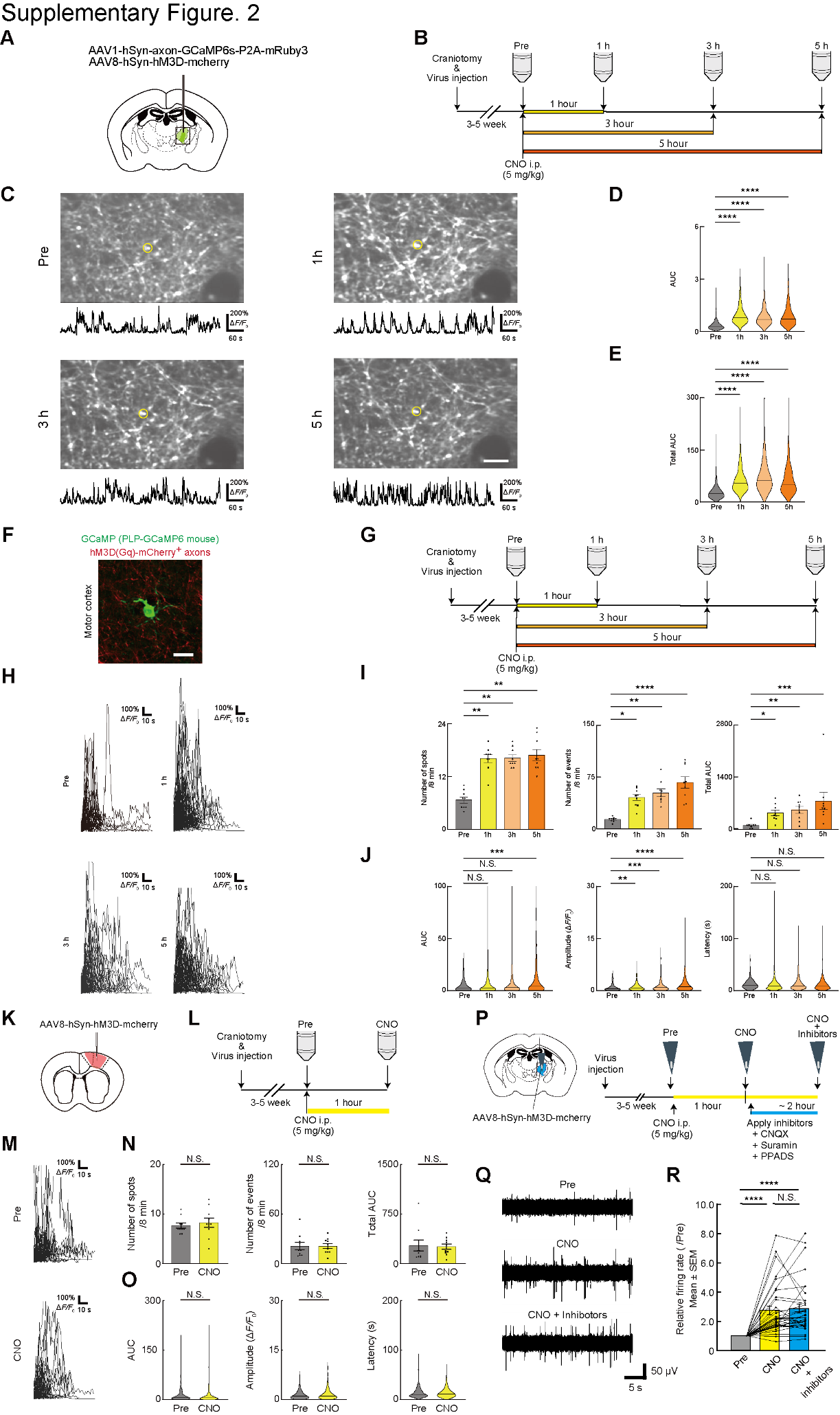


**Supplementary Figure 2.** Chemogenetic activation of ventral-anterior/ventral-lateral thalamic nuclei (VA/VL) neurons promotes VA/VL axons and oligodendrocyte (OC) activity in layer 1 (L1) of motor cortex (M1) for at least 5 hr

(A) Experimental protocol for adeno associated virus (AAV) injection (AAV1-hSyn-axon-GCaMP6s-P2A-mRuby3 and AAV8-hSyn-hM3D(Gq)-mCherry) into VA/VA neurons.

(B) Two-photon Ca^2+^ imaging was performed 3–5 weeks after surgical operation (AAV injection and craniotomy [first]). A synthetic ligand clozapine *N*-oxide (CNO) was administered for hM3D activation (5 mg/kg, i.p.) after Pre-imaging (Pre). After 1, 3, and 5 hr following CNO application, two-photon Ca^2+^ imaging of axons in M1 was performed again (1h, 3h, 5h). Two-photon Ca^2+^ imaging was obtained from the same axons in all imaging sessions.

(C) Representative images and Ca^2+^ traces of axons projecting from the VA/VL to M1 during activation of VA/VL neurons with CNO injection. Scale bar, 10 µm.

(D, E) The AUC and total AUC of axonal Ca^2+^ responses were significantly increased with CNO injection and that persisted for 5 hr. *****P* < 0.0001, Kruskal-Wallis test followed by Dunn’s test. Violin plots show median (black line) and distribution of the data. For detailed data, check the source data file.

(F) Typical image of axons projecting from VA/VL to M1 and OCs in M1. Scale bar, 20 µm.

(G) Experimental protocol of Ca^2+^ imaging in OCs during VA/VL neuronal activation with CNO injection.

(H) Representative Ca^2+^ traces from spots of typical GCaMP’+ve cells at before (Pre) and 1, 3, and 5 hr after CNO injection.

(I) Ca^2+^ spots, Ca^2+^ events and total area under the curve (AUC) were significantly increased after CNO application for at least 5 hr. N.S., not significant, **P* < 0.05, ***P* < 0.01, ****P* < 0.001, *****P* < 0.0001, Kruskal-Wallis test followed by Dunn’s test. Data are presented as mean ± standard error of mean. For detailed data, check the source data file.

(J) No statistically significant differences were detected in AUC (1 hr and 3 hr after CNO injection) and Latency between Pre-imaging and after CNO injection. Amplitude and AUC (5h after CNO) was significantly increased after CNO application. N.S., not significant, ***P* < 0.01, ****P* < 0.001, *****P* < 0.0001, Kruskal-Wallis test followed by Dunn’s test. Violin plots show median (black line) and distribution of the data. For detailed data, check the source data file.

(K) Experimental protocol for AAV injection (AAV8-hSyn-hM3D(Gq)-mCherry) into M1.

(L) Two-photon Ca^2+^ imaging was performed 3–5 weeks after surgical operation (AAV injection and craniotomy [first]). CNO was administered for hM3D activation (5 mg/kg, i.p.) after Pre-imaging (Pre). Two-photon Ca^2+^ imaging of OC in M1 was performed again 1 hr after CNO application. Two-photon Ca^2+^ imaging of OCs was obtained from the same cells in all imaging sessions.

(M) Representative Ca^2+^ traces from spots of typical GCaMP’+ve cells at before (Pre) and 1 hr after CNO injection.

(N) No statistically significant differences were detected in Ca^2+^ spots, Ca^2+^ events and total AUC during activation of cortical neurons in M1. N.S., not significant, Mann Whitney U test. Data are presented as mean ± standard error of mean. For detailed data, check the source data file.

(O) No statistically significant differences were detected in the AUC, Amplitude, and Latency during activation of cortical neurons in M1. N.S., not significant, Mann Whitney U test. Violin plots show median (black line) and distribution of the data. For detailed data, check the source data file.

(P) Experimental protocol for recording VA/VL neuron activity during chemogenetic activation with neurotransmitter receptor antagonists: 6-cyano-7-nitroquinoxaline-2,3-dione disodium (CNQX), Suramin hexasodium salt (Suramin), and pyridoxalphosphate-6-azophenyl-2',4'-disulfonic acid tetrasodium salt (PPADS).

(Q) Typical extracellular recordings from VA/VL neurons baseline (Pre), during chemogenetic activation before (CNO), and after the application of CNQX, Suramin and PPADS (CNO + inhibitors).

(R) There was no significant change in the activity of VA/VL neurons activated by chemogenetic method before and after the application of these neurotransmitter receptor antagonists. ****P < 0.0001, N.S., not significant, Friedman test followed by Dunn’s test. For detailed data, check the source data file.


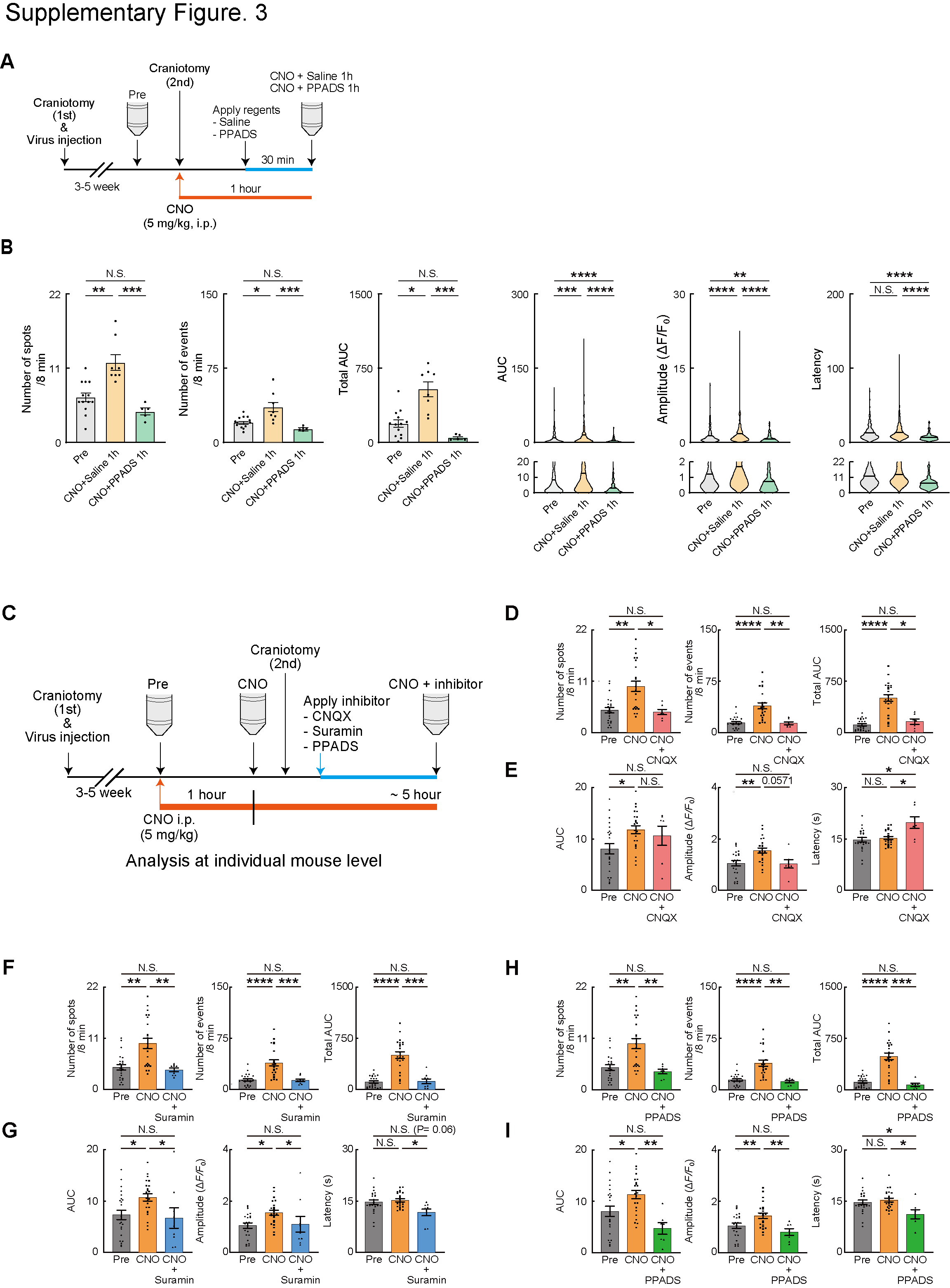

**Supplementary Figure 3.** One hr chemogenetic activation of ventral-anterior/ventral-lateral thalamic nuclei (VA/VL) neurons enhanced oligodendrocyte (OC) activity in L1 of M1 and neuronal activity-dependent Ca^2+^ responses in OCs at individual mouse level before and after application of neurotransmitter receptor antagonists

(A) To verify that the effect of antagonists was not due to the difference in the time course after chemogenetic activation, this experiment (the chemogenetic activation of the thalamocortical circuit and two-photon Ca^2+^ imaging with the application of saline or pyridoxalphosphate-6-azophenyl-2’,4’-disulfonic acid tetrasodium salt (PPADS) 1 hr after CNO activation) was performed. AAV8-hSyn-hM3D(Gq)-mCherry vector was injected into the VA/VL neurons and craniotomy (first) was performed. Then, 3–5 weeks after surgical operation (AAV injection and craniotomy [first]), two-photon Ca^2+^ imaging was performed. CNO was administered for hM3D activation (5 mg/kg, i.p.) after Pre-imaging (Pre). After Pre-imaging, saline or PPADS was applied on the brain surface by craniotomy (second), followed by second imaging (CNO + saline or PPDAS) 1 hr after CNO application.

(B) Ca^2+^ responses (Ca^2+^ spots, Ca^2+^ events, total area under the curve [AUC], AUC, Amplitude and Latency) in OC persisted in the saline group. In contrast, Ca^2+^ responses in OC significantly reduced in the PPADS group.

(C, D, F, H) Ca^2+^ spots, Ca^2+^ events and total area under the curve (AUC) before and after CNO application at individual mouse level. N.S., not significant, **P* < 0.05, ***P* < 0.01, ****P* < 0.001, *****P* < 0.0001, Kruskal-Wallis test followed by Dunn’s test. Data are presented as mean ± standard error of mean. For detailed data, check the source data file.

(C, E, G, I) AUC, Amplitude and Latency before and after CNO application at individual mouse level. N.S., not significant, **P* < 0.05, ***P* < 0.01, Kruskal-Wallis test followed by Dunn’s test. Violin plots show median (black line) and distribution of the data. For detailed data, check the source data file.


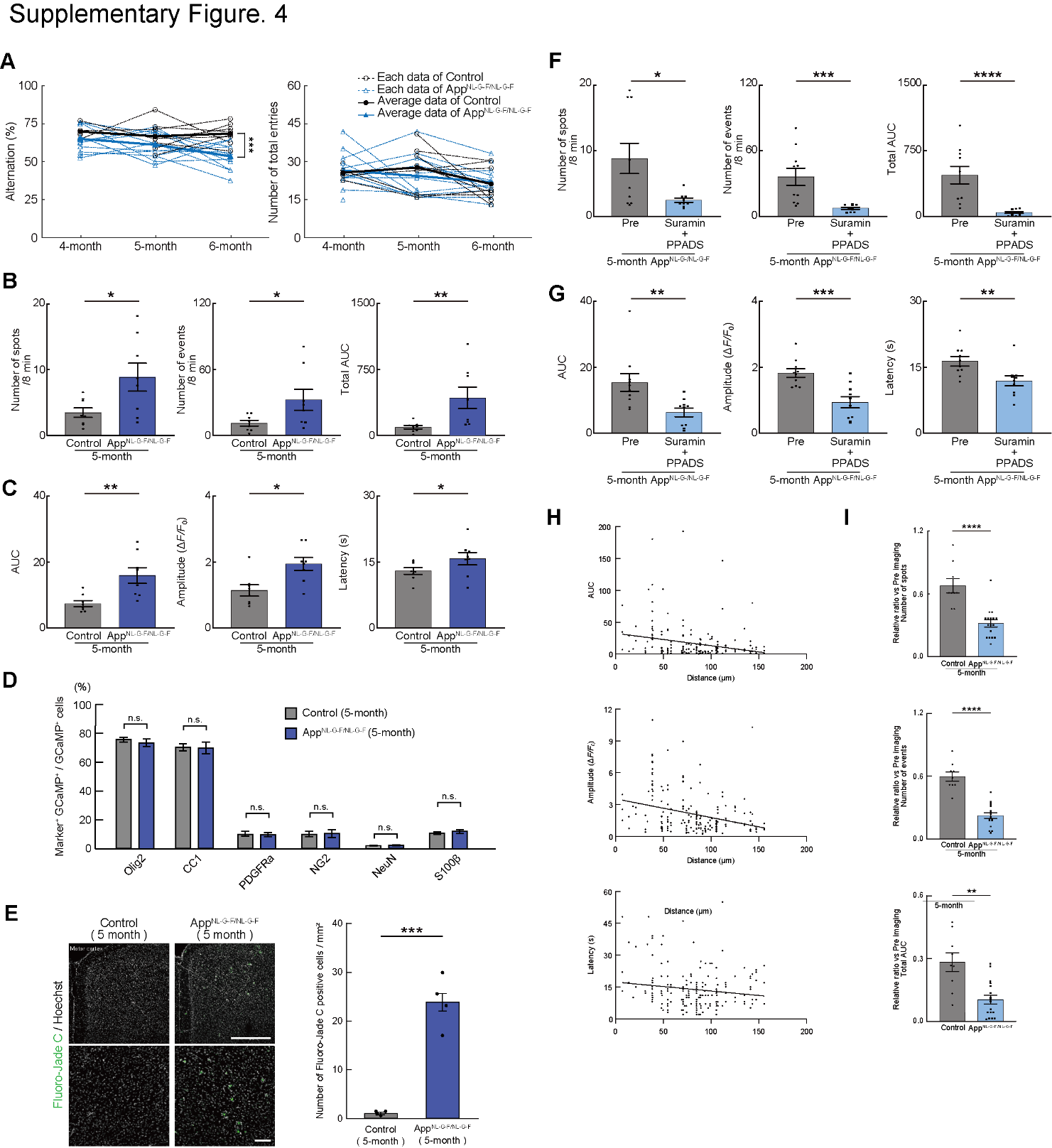


**Supplementary Figure 4.** Ca^2+^ responses of oligodendrocytes (OCs) in 5-month-old App^NL-G-F/NL-G-F^ mice was facilitated by adenosine triphosphate (ATP) signals

(A) Memory impairment in App^NL-G-F/NL-G-F^ mice. The Y-maze test was performed at 4-, 5-, and 6-months old mice. Left: Alternation was significantly decreased at 6 months of age in App^NL-G-F/NL-G-F^ mice compared with control mice. No statistical differences were detected in Alternation at 4 and 5 months of age between App^NL-G-F/NL-G-F^ and control mice. ****P* < 0.001, by Mann Whitney U test. For detailed data, check the source data file.

(B) Even at individual mouse level, Ca^2+^ spots, Ca^2+^ events and total area under the curve (AUC) were significantly higher in App^NL-G-F/NL-G-F^ mice than in control mice at 5 months of age. **P* < 0.05, ***P* < 0.01, Mann Whitney U test. Error bar shows mean ± standard error of mean. For detailed data, check the source data file.

(C) Even at individual mouse level, AUC, Amplitude, and Latency were significantly higher in App^NL-G-F/NL-G-F^ mice than in control mice at 5 months of age. **P* < 0.05, ***P* < 0.01, Mann Whitney U test. Error bar shows mean ± standard error of mean. For detailed data, check the source data file.

(D) Quantification of GCaMP expression in the motor cortex of 5-month-old App^NL-G-F/NL-G-F^ and age-matched control mice and co-localization with markers for OC + oligodendrocyte precursor cell (OPC) (Olig2), OC (CC1), OPC (PDGFRα, NG2), neuron (NeuN), astrocyte (S100β). For detailed data, check the source data file.

(E) Typical images of Fluoro-Jade C staining from 5-month-old App^NL-G-F/NL-G-F^ and age-matched control mice. There was a significant difference in the number of dying or dead cells between 5-month-old App^NL-G-F/NL-G-F^ and age-matched control mice. ****P* < 0.001, Unpaired *t*-test. Error bar shows mean ± standard error of mean. Scale bars: in (top), 500 μm; in (bottom), 100 μm. For detailed data, check the source data file.

(F) Ca^2+^ spots, Ca^2+^ events and total AUC were significantly decreased after Suramin + PPADS application at individual mouse level. **P* < 0.05, ****P* < 0.001, *****P* < 0.0001, Mann Whitney U test. Data are presented as the mean ± standard error of mean. For detailed data, check the source data file.

(G) AUC, Amplitude and Latency were significantly decreased after Suramin + PPADS application at individual mouse level. ***P* < 0.01, ****P* < 0.001, Mann Whitney U test. Data are presented as the mean ± standard error of mean. For detailed data, check the source data file.

(H) Scatter plot of distances between the Ca^2+^ spots in OC and Aβ deposition. There is no correlation in the distance between the Ca^2+^ spots and Aβ deposition. For detailed data, check the source data file.

(I) ATP inhibitors (Suramin + PPADS) suppressed the Ca^2+^ responses in OC of 5-month-old App^NL-G-F/NL-G-F^ mice more strongly than that of age-matched control mice. ***P* < 0.01, *****P* < 0.0001, Mann Whitney U test. Data are presented as the mean ± standard error of mean. For detailed data, check the source data file.
